# A global database of diversified farming effects on biodiversity and yield

**DOI:** 10.1038/s41597-021-01000-y

**Published:** 2021-08-10

**Authors:** Sarah K. Jones, Andrea C. Sánchez, Stella D. Juventia, Natalia Estrada-Carmona

**Affiliations:** 1 Bioversity International, Parc Scientifique Agropolis II, 34397 Montpellier, France; 2 Farming Systems Ecology Group, Wageningen University & Research, 6700 AK Wageningen, The Netherlands

**Keywords:** Agriculture, Agroecology

## Abstract

With the Convention on Biological Diversity conference (COP15), United Nations Climate Change Conference (COP26), and United Nations Food Systems Summit, 2021 is a pivotal year for transitioning towards sustainable food systems. Diversified farming systems are key to more sustainable food production. Here we present a global dataset documenting outcomes of diversified farming practices for biodiversity and yields compiled following best standards for systematic review of primary studies and specifically designed for use in meta-analysis. The dataset includes 4076 comparisons of biodiversity outcomes and 1214 of yield in diversified farming systems compared to one of two reference systems. It contains evidence from 48 countries of effects on species from 33 taxonomic orders (spanning insects, plants, birds, mammals, eukaryotes, annelids, fungi, and bacteria) of diversified farming systems producing annual or perennial crops across 12 commodity groups. The dataset presented provides a resource for researchers and practitioners to easily access information on where diversified farming systems effectively contribute to biodiversity and food production outcomes.

## Abstract (summary)

**Table:** 

Measurement(s)	yield trait • biodiversity outcomes
Technology Type(s)	Meta-Analysis
Factor Type(s)	Crop type • non-domesticated taxa
Sample Characteristic - Organism	Mammalia • Arthropoda • Aves • Amphibian • Annelida • Eukaryota • Nematoda • Plant • Reptile
Sample Characteristic - Environment	agricultural field • natural environment
Sample Characteristic - Location	global

Machine-accessible metadata file describing the reported data: 10.6084/m9.figshare.14723913

## Background & Summary

Our food systems need to be transformed to provide nutritious food for the world’s growing population while halting and reversing pressure on local and global biodiversity and natural resources^1–3^. Diversified farming systems are at the core of agroecology, which has been proposed as a way of achieving sustainable food systems^4–8^. They encompass a range of practices that enhance agrobiodiversity across spatial or temporal scales^9^. Practices may involve increasing the richness or evenness of crop cultivars or species at plot, field or farm level, e.g. through crop rotations, cover crops, intercropping, and agroforestry (a type of intercropping with woody plants); integrating livestock and fish production with crop production, or; better embedding natural or semi-natural vegetation into fields and farms, e.g. using hedgerows, set-aside, fallow fields and insectary strips^10,11^.

Evidence is building that diversified farming systems have positive impacts on biodiversity and a multitude of ecosystem services and does not necessarily come at the cost of yields or farm profitability^10,12,13^. Diversifying can have economic benefits by reducing input requirements and increasing or stabilising yields^10,14–17^ including for national food production^18^. Previous reviews that have considered biodiversity and yield outcomes simultaneously tend to pool species into broad taxonomic groups and do not always record agronomic factors (crop type, tillage, agrochemical use). This makes it difficult to use the information to understand what crop combinations, farm management and landscape contexts make diversified farming systems more likely to provide positive outcomes for biodiversity and yields. Yet this is the level of information that is needed for interventions at the field and farm level.

**Fig. 1:**
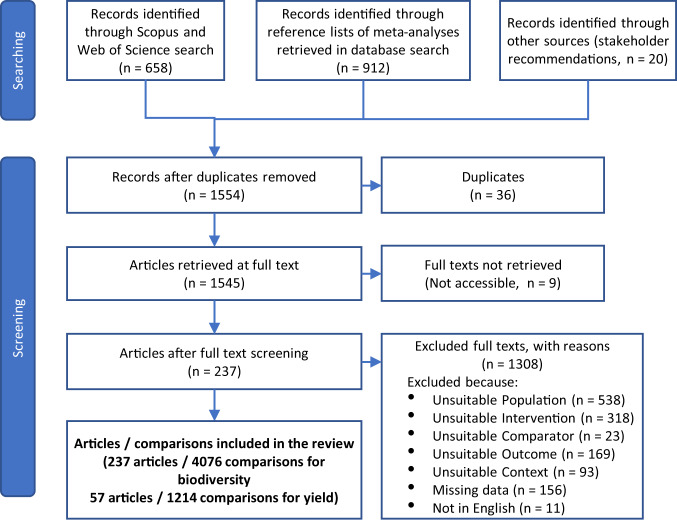
Prisma flowchart of the literature search and screening process.

**Fig. 2:**
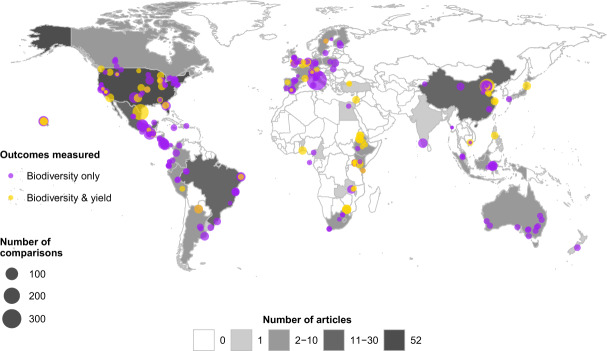
Global distribution of articles and comparisons for each outcome measured.

**Fig. 3:**
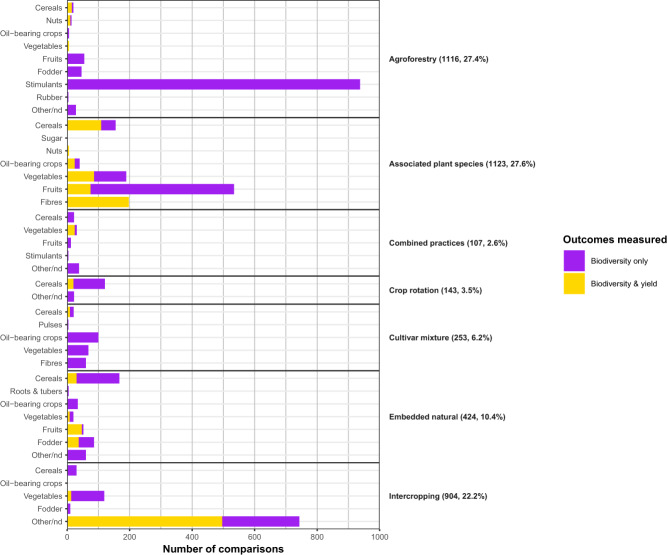
Distribution of comparisons by main crop type grouped according to the Food and Agriculture Organisation of the United Nations (FAO) commodity groups and diversified farming system, distinguishing the outcome measured. Numbers in parentheses adjacent to each diversified farming system indicate the number and percentage of comparisons.

We published and implemented a protocol for conducting a systematic review of peer-reviewed literature to compare outcomes for biodiversity and agronomic yields in diversified farming systems, compared to simplified farming systems or (for biodiversity outcomes only) natural habitat^19^. The resultant global dataset contains 4076 comparisons of biodiversity outcomes from 237 articles (Fig. 1), of which 30% (1214) from 57 articles also contain information on yield outcomes. Of the total comparisons, 82% compare outcomes of diversified farming interventions (also called the treatment) using simplified farming systems as the comparator (also called the control or reference system), while 18% use natural habitat as the comparator. Comparisons were obtained from 48 countries across 6 continents, with nearly a quarter of these from the USA (22%), 11% from Mexico, 10% from China, 9% from Italy, 5% Brazil, 5% Costa Rica, while other countries each represented <5% of comparisons (Fig. 2). In terms of the diversified farming practices, agroforestry is the most well represented (27% of comparisons), followed by intercropping (22%) while combined practices such as integrated crop-livestock systems are the least well represented constituting 2.6% of comparisons. For biodiversity, arthropods represent 69% of comparisons, followed by plants (12%) and birds (6%), while mammals, reptiles, amphibians, nematodes and other taxonomic groups each constitute <5% of comparisons. These taxa were classified into eleven different functional groups, with crop pests and their natural enemies the best represented constituting respectively 32% and 27% of comparisons (Table 1). In total, 38% of comparisons are from plots where cereals or stimulants were the main crop in the diversified farming system, while 31% represent fruits or vegetables. Some other crop groups including roots and tubers, pulses, and nuts, are less well studied, each representing <1% of comparisons (Fig. 3).

**Table 1:** Number of comparisons of biodiversity outcomes by taxonomic and functional group, and in parentheses number of comparisons with both biodiversity and yield outcomes.

Taxanomic group	Autotrophs	Decomposers	Frugivores	Granivores	Herbivores	Insectivores	Natural enemies	Omnivores	Other	Pests	Pollinators	Total
Amphibians	0	0	0	0	0	0	34	0	10	0	0	44
Annelida	0	79 (9)	0	0	0	0	0	0	0	0	0	79 (9)
Arthropoda	0	137 (8)	0	0	243 (35)	0	936 (330)	0	112 (35)	1121 (573)	275 (19)	2824 (1000)
Birds	0	0	17 (6)	17 (6)	0	13	112 (12)	17 (6)	69 (3)	0	5	250 (33)
Eukaryotes	0	75 (34)	0	0	0	0	0	0	0	0	0	75 (34)
Fungi	0	92 (36)	0	0	0	0	0	0	0	9 (9)	0	101 (45)
Mammals	0	0	11	0	0	92	12	0	70 (1)	1 (1)	1	187 (2)
Nematoda	0	0	0	0	0	0	0	0	0	16	0	16
Plantae	334 (2)	0	0	0	0	0	0	0	2	149 (89)	0	485 (91)
Reptiles	0	0	0	0	0	7	8	0	0	0	0	15
Total	334 (2)	383 (87)	28 (6)	17 (6)	243 (35)	112	1102 (342)	17 (6)	263 (39)	1296 (672)	281 (19)	4076 (1214)

The dataset represents the most comprehensive quantitative evidence base documenting the trade-offs and synergies of farm diversification for yield and biodiversity outcomes, across different production systems and geographies. It highlights a global shortage of primary studies on diversified farming system outcomes for: mammals, reptiles, and amphibians; in plots growing roots, tubers, pulses, nuts, and marginal cereal, vegetable and fruit crops, or practicing integrated crop-fish or crop-livestock farming, and; in northern Africa, southwest Asia, and Eastern Europe. It shows a lack of articles reporting both biodiversity and yield outcomes, particularly for coffee, cocoa and several fruit and vegetable crops, while a high proportion did not specify agrochemical or soil management practices at the study sites. This emphasizes the need for agronomic management factors and outcomes to be more systematically captured in ecological studies, and vice-versa, to enable trade-off analyses. Further analysis using this dataset can contribute to closing a critical data gap in the evidence base available to policymakers and investors on how to sustainably transform our food production systems through diversifying farming systems. Additionally, the dataset can be used to shape future research agendas towards collecting data across crops, geographies, practices and functional or taxonomic groups poorly represented in the current body of knowledge.

## Methods

The data collection protocol is described in Sánchez *et al*.^19^ and followed Reporting Standards for Systematic Evidence Syntheses (ROSES) guidelines^20^. Philibert *et al*.^21^, also proposed eight criteria for conducting high quality meta-analysis, which overlap to some extent with ROSES guidelines. Our methods fulfil the Philibert *et al*. requirement to use a repeatable procedure for paper selection, provide a list of references, and ensure availability of the dataset, while other quality criteria are only relevant at the meta-analysis stage.

### Search process

The literature search was conducted on 29 November 2019 and updated on 5 January 2021, and aimed to identify relevant English language articles published in peer-reviewed literature. We searched in titles, abstracts and keyword lists of literature in the Scopus and Web of Science databases, using the following search string (formatted for Scopus; see Dataset 1 for the equivalent string formatted for Web of Science): TITLE-ABS-KEY (“agricultur*” AND “biodiversity”) AND TITLE-ABS-KEY (“agro?ecology” OR “agro?biodivers*” OR “agroforestry” OR “border plant*” OR “riparian buffer” OR “woodlot” OR “hedgerow” OR “cover crop*” OR “crop rotation” OR “crop divers*” OR “inter?crop*” OR “mixed crop*” OR “cultivar mixture” OR “plant divers*” OR “polyculture” OR “tree divers*” OR “variet* diversity” OR “fallow” OR “field margin*” OR “grass strip*” OR “*flower strip*” OR “insect* strip” OR “conservation strip” OR “vegetation strip” OR “catch crop” OR “inter?crop*” OR “crop variety” OR “crop sequenc*” OR “mixed farming” OR “land sparing” OR “landscape heterogeneity” OR “heterogeneous landscape” OR “landscape diversi*” OR “divers* landscape” OR “homogeneous landscape” OR “landscape homogeneity” OR “landscape complexity” OR “simplif* landscape” OR “complex landscape” OR “multi?function* landscape” OR “integrated crop-livestock” OR “integrated crop-forest” OR “land sharing”) AND TITLE-ABS-KEY (“ richness” OR “ abundance” OR “species diversity” OR “functional diversity” OR “index”) AND TITLE-ABS-KEY (“crop yield” OR “crop production”) AND (LIMIT-TO (LANGUAGE, “English”)). We extracted the primary studies included in all relevant meta-analyses identified from the database search. In addition, we included a small number of peer-reviewed articles known to scientists consulted through the Sustainable Foods project and which were not retrieved by the search string or from previous meta-analyses. In total, 1590 articles with the potential to be included in the meta-analysis were identified (Fig. 1).

### Article screening

All identified articles were screened at full-text level. We used the PICOC (Population, Intervention, Comparator, Outcomes, Context) framework to define the inclusion-exclusion criteria as described in Sánchez *et al*.^19^. These criteria required that, to be included: (i) the article presents a quantitative comparison of a diversified farming system (Intervention) compared to either a relatively simplified farming system (first Comparator) or to natural habitat (second Comparator), ii) the article reports quantitative outcomes for any terrestrial organism that is non-domesticated (Population), iii) the article provides the mean or median, variance and sample size for biodiversity outcomes, and outcome measures in comparator and intervention sites were collected using comparable sampling approaches (Outcome), iv) results are from primary field studies and not from experiments conducted in greenhouses or laboratories (Context).

Diversified farming systems were defined as agricultural plots where: i) more than one plant species or variety is cultivated at multiple temporal and/or spatial scales, such as crop rotations, intercropping or agroforestry, or ii) semi-natural habitat such as hedgerows and flower strips is embedded into the system, or iii) crop production is integrated with livestock or fish production, such as aquaculture or integrated crop-livestock systems. Simplified farming systems were agricultural plots with less diversity than in eligible interventions, *i.e*., plots with relatively fewer plant species or varieties (usually monocultures), less semi-natural habitat embedded, or no mixed crop-animal production. Where natural habitat was used as a comparator, this was defined as habitat that is not actively used for human activities, such as primary and secondary forests, wetlands, unmanaged grasslands and shrublands.

Suitable outcome metrics for biodiversity included any comparable quantified measure, such as richness, abundance, or Shannon’s diversity index. While studies only needed to report biodiversity outcomes to be considered for inclusion, we recorded harvested yield in all cases where this was reported and met our inclusion criteria. For yield outcomes to be included, the article must have provided means or medians, variance and sample sizes, and outcome measures at intervention and comparator sites must have been collected using comparable sampling approaches. Suitable outcome metrics for yields included the land equivalent ratio, weight of harvested produce per unit land area, or counts of harvested produce per standardized unit (e.g. grape bunch per plant, apples per branch). For comparisons comparing intercropped or agroforestry systems against simplified farming systems, the land equivalent ratio was prioritized as the outcome metric while other metrics were used only when the land equivalent ratio could not be calculated.

In total, 237 (14.9%) of retrieved articles met our inclusion criteria (Fig. 1).

### Data extraction

From each article that met our inclusion criteria, we extracted qualitative data on: the literature source (e.g. authors, publication year, title); crop type (common name, scientific name); agricultural system (e.g. intercropping, monoculture, agroforestry, integrated crop-livestock system, crop rotation, set aside); non-domesticated taxa sampled (common and scientific names); functional group of the non-domesticated taxa, if specified (e.g. pest, decomposer, predator); biodiversity outcome metric (e.g. species richness, abundance, Shannon’s diversity index); yield metric (e.g. kilogram per hectare, grams per plant, land equivalent ratio); sampling method used (e.g. transect, trap); pesticide use (yes or no, and kg/ha); fertilizer use (yes or no, and chemical fertiliser use yes or no); soil management (e.g. tillage, no tillage, slash and burn); landscape characteristics (e.g. % agricultural land use, climate); and study location (local name, country and geographic coordinates). Following initial data-entry, we classified several variables into categories to facilitate data exploration and analysis. This included categorizing crops by the Food and Agriculture Organisation of the United Nations commodity group, woodiness (e.g. tree, shrub, herb), and growth cycle (perennial, annual), and documenting the phylum, class, order and functional group of each non-domesticated taxon.

We extracted quantitative data on: biodiversity outcome means or medians, variance and sample size; yield means or median, variance and sample size; farm size, if specified; length of time that the land has been in its current state, and; sampling duration (in days, from start to finish). Data on biodiversity outcomes and yield were extracted from figures using GetData Graph Digitizer 2.26 or WebPlotDigitizer v4.2. Where outcome values or units in an article were unclear or not provided, the corresponding author was contacted by email to request this information. If the author did not respond, the data entry was removed. We provide a dictionary of how the extracted data were recorded and coded in Dataset 2.

Data were organized using R-4.0.0 (R-Core Team, 2013) such that each row contained a pair of biodiversity outcomes and, where provided, a pair of yield outcomes, for a single comparator-intervention pair. In total, 237 studies containing 4076 comparisons of biodiversity outcomes and 1214 comparisons of yield outcomes were retained for analysis (Fig. 1).

## Data Records

The data described in this article are available in spreadsheet format on *Harvard DataVerse* at 10.7910/DVN/XIDI1X^22^, and include:

- Dataset_1_sources: Search strings used in Web of Science and Scopus together with a list of literature retrieved during all search phases and their eligibility for inclusion in the meta-analysis. The dataset includes reasons for exclusion.

- Dataset_2_outcomes: Data (mean or median, variance and sample sizes) extracted from primary studies on biodiversity and yield outcomes for each comparator-intervention pair, where diversified farming systems are the intervention and either simplified farming systems or natural habitat are the comparator. The dataset includes associated contextual data such as crop or natural habitat type, crop management, agrochemical use, site location, and sampling method (Data tab). The dataset includes rules for entering data systemically (Data dictionary tab) and reclassifying raw data into standardised groups for analysis (Key tab).

## Technical Validation

All data entries were double checked by a second reviewer. We conducted an internal validity assessment to identify possible sources of bias. For the assessment, each comparison of effects on biodiversity or yield outcomes (hereafter referred to as effect sizes) was assessed against five criteria aimed to capture potential sources of internal bias (Table 2). In total, over half (54.2%) of biodiversity effect sizes met all five of the quality criteria, while 23.3% failed to meet one or more of these criteria mainly due to 19.1% of comparisons with a small sample size. For yield effect sizes, 41.8% met all five of the criteria, while 33.2% did not, driven by small sample sizes used to collect yield data in approximately half of primary studies. Comparisons between diversified farming systems and simplified farming systems or natural habitats were conducted at sites less than 1.1 km apart in 92.1% of cases, and were therefore likely to have similar site characteristics for more meaningful comparisons. Approximately three-quarters of comparator and intervention sites had been in their current state for at least 1 year (77.8% and 77.5% respectively), increasing the likelihood that outcomes are a result of current and not previous habitat and management practices.

**Table 2:** Percentage of comparisons meeting each quality criteria, based on the internal validity assessment.

Criteria	% comparisons that meet the criteria	% comparisons that do not meet the criteria	% comparisons that could not be assessed (no data)
A. ≥ 1 yr in current state for comparator	77.8	3.8	18.3
B. ≥ 1 yr in current state for intervention	77.5	3.6	18.9
C. ≥ 5 biodiversity samples for comparator	80.9	19.1	0
D. ≥ 5 biodiversity samples for intervention	80.9	19.1	0
E. ≥ 5 yield samples for comparator	50.4	49.6	0
F. ≥ 5 yield samples for intervention	51.2	48.8	0
G. Similar site characteristics for comparator and intervention	92.1	4.8	3.1
All criteria for yield effects (A-B,E-G)	41.8	33.2	25
All criteria for biodiversity effects (A-D,G)	54.2	23.3	22.5

## Data Availability

Code used in processing the data are available on Github at the following link: https://github.com/skatejones/SustainableFoods.
